# Update on In Vitro Diagnostic Tools and Treatments for Food Allergies

**DOI:** 10.3390/nu15173744

**Published:** 2023-08-26

**Authors:** Mariano Brasal-Prieto, Laura Fernández-Prades, Hala Dakhaoui, Francisco Sobrino, Soledad López-Enríquez, Francisca Palomares

**Affiliations:** Department of Medical Biochemistry, Molecular, Immunology, School of Medicine, University of Seville, Av. Sanchez Pizjuan s/n, 41009 Seville, Spain; marbrapri123@gmail.com (M.B.-P.); laurafprades@gmail.com (L.F.-P.); hala.bouita@hotmail.com (H.D.); fsobrino@us.es (F.S.)

**Keywords:** food allergy, immunology, immunotherapy, nanostructures, probiotic, herbal medicine

## Abstract

Food allergy (FA) is an adverse immunological reaction to a specific food that can trigger a wide range of symptoms from mild to life-threatening. This adverse reaction is caused by different immunological mechanisms, such as IgE-mediated, non-IgE-mediated and mixed IgE-mediated reactions. Its epidemiology has had a significant increase in the last decade, more so in developed countries. It is estimated that approximately 2 to 10% of the world’s population has FA and this number appears to be increasing and also affecting more children. The diagnosis can be complex and requires the combination of different tests to establish an accurate diagnosis. However, the treatment of FA is based on avoiding the intake of the specific allergenic food, thus being very difficult at times and also controlling the symptoms in case of accidental exposure. Currently, there are other immunomodulatory treatments such as specific allergen immunotherapy or more innovative treatments that can induce a tolerance response. It is important to mention that research in this field is ongoing and clinical trials are underway to assess the safety and efficacy of these different immunotherapy approaches, new treatment pathways are being used to target and promote the tolerance response. In this review, we describe the new in vitro diagnostic tools and therapeutic treatments to show the latest advances in FA management. We conclude that although significant advances have been made to improve therapies and diagnostic tools for FA, there is an urgent need to standardize both so that, in their totality, they help to improve the management of FA.

## 1. Introduction

Food allergy (FA) is an abnormal and exacerbated response of the immune system to certain food allergens by immunoglobulin E (IgE)-mediated [1], non-IgE-mediated or mixed reaction [2]. The mechanisms involved consist of the dysregulated immune responses and a skewing towards a type 2 immune response. This is associated with production of IgE antibodies and inflammatory cytokines, which are reviewed in detail in the next section. This immune response can result in various ways towards allergen foods, leading to a wide variety of symptoms and clinical manifestations.

The food allergens that most frequently cause the allergies are the following: cow’s milk, eggs, fish, peanuts, peach, and soy amongst others [3]. FA patients can suffer a wide variety of symptoms ranging from mild to severe, such as effecting the digestive system, skin, respiratory tract and in extreme cases it can cause a severe allergic reaction known as anaphylaxis [4]. However, some severe reactions can also be associated with other risk factors (co-factors) such as exercise, nonsteroidal anti-inflammatory drugs (NSAIDs), alcohol, alterations in the intestinal microbiota, genetic and even environmental factors. Theses underlying mechanisms are unknown [5,6], which represents a significant burden for health and quality of life.

FA in our society is growing, already affecting an estimated 1 to 4% of young children, 9 to 11% of adults from Europe and USA, respectively [7]. It is believed that several co-factors may be contributing to this increase.

FA diagnosis can be complex due to the nature of the immunological mechanisms involved in allergic reactions to foods. Therefore, the need to improve strategies for diagnosis and therapy is essential. Diagnosis of FA relies on the combination of clinical/reaction history, skin and IgE testing as well as oral food testing. This is currently favored by method of using in vitro diagnostic techniques such as the basophil activation test (BAT) or the mast cell activation test (MAT) [8] which complement the diagnosis.

In recent years, important advances have been made in the search for other options for the treatment of FA, mainly emphasizing immunomodulatory therapies such as specific allergen immunotherapies (AIT). For example, oral immunotherapy (OIT) using peanut (Arachis hypogea) allergen powder-dnfp (PTHA) in children with peanut allergy has been approved as a treatment in certain cases [9]. Although AIT has achieved a beneficial response for allergic patients, it does not cure the disease so avoiding foods that may contain allergens strictly continues to be applied. Advances in the field of AIT and other novel therapies (nanoparticle design, probiotics, symbiotic, and herbal extracts) have emerged as new options for the development of FA treatments, including the use on monoclonal antibody (anti-IgE). In this review, we provide an overview of the most recent major advances in the diagnosis (in vitro tools) and FA treatment.

## 2. Immune Mechanism in FA

In FA, a physio-pathological reaction of the immune system is triggered by the ingestion of a food protein or food allergens. This leads to type I hypersensitivity and immediate reaction, which involves IgE-mediated release of antibodies against the soluble antigen. Theses reactions can be IgE-mediated, non-IgE-mediated, and mixed IgE reactions [10,11]. IgE-mediated FA reactions develop a multi-organ system anaphylaxis. Non-IgE-mediated FAs include a group of disorders characterized by subacute or chronic inflammatory processes affecting the gastrointestinal tract [12]. Mixed IgE and non-IgE-mediated reactions such as food protein-induced allergic proctocolitis, food protein-induced enterocolitis or eosinophilic gastrointestinal disorders [13] have variable symptoms.

### 2.1. IgE-Mediated Reactions

**Figure 1:**
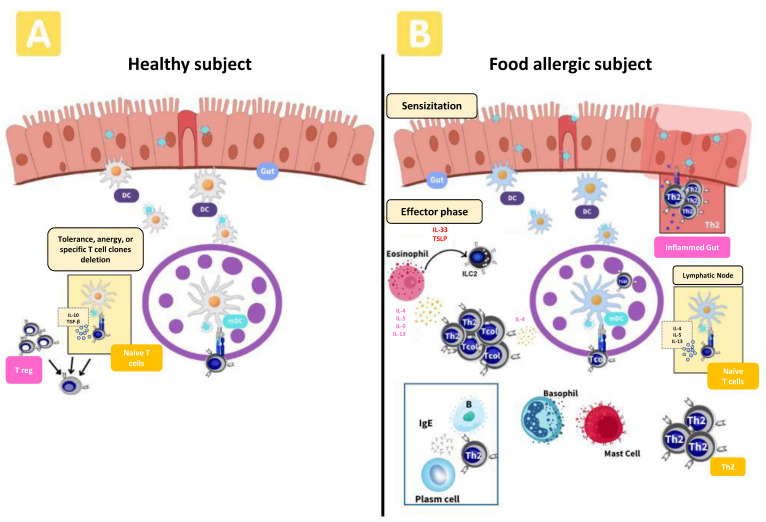
Immune cells involved in healthy subject’s tolerance to food allergens (**A**) and with a food allergic subject with IgE-mediated reactions (**B**).

Sensitization to food allergens occurs in subjects after intake of foods which leads to an adverse inflammatory immune response [14]. The sensitization occurs in a microenvironment that shows damage to the epithelial permeability and dysbiosis (studied in Section 5.1.) which contributes to an imbalance of the host metabolism and immunometabolism. In response, allergens are taken up by dendritic cells (DCs) which interact with a naïve T cell and stimulate the effector response. In this process, DCs undergo phenotypic changes by increasing surface costimulatory receptors and cytokine production to induce a Th2 response (Figure 1) [15].

Recent studies have demonstrated the involvement of innate lymphoid cells (ILCs) emerging as a key in the cause of FA [16,17]. Among them, ILC2 are activated by interleukin (IL)-25, IL-33, thymic stromal lymphopoietin (TSLP) originates from their expansion and production of Th2 cytokines (IL-4, IL-5, and IL-13) [18]. Higher levels of these cytokines have been observed in FA patients [19]. With IL-33, after inducing the ILC2 activation, it produces high IL-4 levels which promotes a Th2 response and can inhibit the regulatory T cell (Treg) function in epithelia (skin and intestinal mucosa) [19]. Therefore, both Th2 and ILC2 release type 2 cytokines that promote B cell differentiation into allergen-specific IgE producing plasma cells which bind to the high-affinity IgE receptor (FcεRI) [11,19]. Moreover, in vitro assays had shown that both IL-33 and IgE-mediated activation of mast cells inhibited the generation of a Treg response pattern from T cells in FA an animal model [20].

In the effector phase, re-exposure to the food allergen triggers rapid IgE-mediated degranulation of mast cells, basophils which release histamine and other inflammatory mediators (PGE2). This is accompanied by the adaptive Th2 response, associated with the manifestation of symptoms and the generation of allergen-specific B and T memory cells [11,19,21]. Additionally, it has recently been proposed that specific effector T cell subsets such as Th1, Th17, Tfh13, Th9 and Th22, might also contribute to ongoing FA [21,22].

### 2.2. Non-IgE-Mediated Reactions

This heterogeneous group of delayed reactions to foods harbor disorders including food protein-induced enterocolitis syndrome (FPIES), food protein-induced allergic proctocolitis (FPIAP), food protein-induced allergic enteropathy (FPE) and food protein-induced dysmotility disorders (gastro-esophageal reflux disorder (GORD) and constipation) [23]. Although the immune mechanism in these disorders remain unclear, they share the characteristics of the complete absence of IgE in the skin serum of patients with FA. Thus, they are being associated generally with a Th2 response pattern such as the classical IgE-mediated FA allergic reactions. In FPES, a pan-leukocyte activation has been observed with association of innate cells and increased gastrointestinal permeability, but the identification of food allergen-specific T lymphocyte is not conclusive [4,24].

### 2.3. Mixed Reactions

Mixed IgE and non-IgE-mediated FAs include eosinophilic gastrointestinal disorders (EGIDs), such as eosinophilic esophagitis (EoE), cow’s and soja’s milk protein allergy (CMPA and SMPA, respectively) and atopic dermatitis [23]. Contrarily to FPIES, in the EoE disorder, T cells may play a central role in the development of this FA, [24]. Moreover, CMPA has been classified as IgE-mediated immediate reaction, non-IgE-mediated delayed reaction, or a mix of both [25,26].

Therefore, understanding the immunological mechanisms that occur in allergic reactions to foods is essential for an accurate diagnosis. The exploration of these mechanisms will allow us to identify the trigger allergen of the reaction, determine the severity of the allergy, amongst others. However, despite the recent studies, we found there are still limitations in the knowledge of the mechanisms, which means that in vitro diagnostic tools still need to be improved as well.

## 3. *FA* In Vitro Supporting Diagnostic Tools

The variability of complex immunological mechanisms contributes to inaccurate diagnosis and complicates the studies on the epidemiology of FA [27,28,29]. The diagnosis of FA is usually made through a combination of the patient’s medical history, SPT, laboratory tests, and/or oral food challenges (OFC). Here, we will review the latest advances in the application of in vitro supporting diagnostic tools, including the current limitations (Table 1).

### 3.1. Allergen-Specific IgE In Vitro Testing

In vitro tests that measure serum sIgE allergen levels are conventionally used to diagnose FA, already being the standardized diagnostic tools [30,31]. The utility of allergen-specific IgE testing as an alternative to the OFC—the diagnostic standard—is being investigated. A study has determined that the combination of the sIgE levels (ImmunoCAP) and the basophil activation test (BAT) to Ses i 1 can decrease the need for OFC in sesame food allergy (SFA) patients. The authors showed Ses i 1 sIgE levels were not robust enough to be used for diagnosis; however, the simultaneous use of BAT and IgE showed positive correlations [32]. Recently, a systematic review using ImmunoCAP demonstrated that Cor a 9 and Cor a 14 drastically improved the specificity of hazelnut allergy diagnosis, compared to hazelnut extract (HE) sIgE. Using a cutoff of 0.35 ku/L, Cor a 14 sIgE had a specificity of 81.7%, compared to 10.8% for HE sIgE, although sensitivity of HE sIgE was slightly superior (95.5% for HE vs. 77.9% for Cor a 14) [33].

Sensitization to allergen components can be detected by using single plex (ImmunoCAP) or multiplex assays (ISAC) [34]. In particular, the multiplex assays offer a complete profile of multiple allergens in a single test. This is especially useful for identifying different allergens present in a patient and better understanding their sensitivities and allergies. A retrospective study for children with a suspected peanut allergy analyzed the Ara h 2 and Ara h sIgE levels using ImmunoCAP and ISAC. The results showed that the determination of Ara h 6 and Ara h 2 sIgE levels in ISAC was considered good predictors of peanut allergy in children. Even so, the levels of Ara h 2 were comparable to the levels obtained with ImmunoCAP. They concluded that the different peanut components using ISAC was an advantage and clinically useful to detect peanut allergic children [35]. It has been published that the sIgE levels measured with ISAC (Act d 1, Act d 2) showed a sensitivity (59.5%) similar to that of ImmunoCAP (with whole kiwi extract, 63.9%). Act d 1 from ISAC was associated with positive sIgE results from whole kiwi extract detected by ImmunoCAP [36], which indicated that the two in vitro tools showed a similar diagnostic capacity. In a Polish study of a large cohort of children determined sIgE to 112 allergen components using ISAC, and sIgE for hazelnut, Cor a 14, Cashew and Ana o 3, using ImmunoCAP. Both in vitro tools determined that Ana o 3 was the allergen component that predicted anaphylaxis. Although its quantity was lower in ISAC compared to ImmunoCAP, due to the sensitivity of sIgE. Despite this, the identification of a single allergen component allowed the authors to determine the risk of severe anaphylaxis in FA [37].

In addition to these in vitro techniques, new systems based on IgE multiplex-immunoblot assay are being evaluated. Recently, a study evaluated improvement of lipid transfers protein syndrome (LTP) diagnosis using a EUROLINE-LTP strip. It was observed that there was a positive correlation between the sIgE levels of the single allergen components (Pru p 3, Mal d 3, Ara h 9, Cor a 8 and Jug r 3) with respect to the sIgE levels of the ImmunoCAP. This new IgE multiplex-immunoblot LTP assay showed a good diagnostic performance allowing the culprit food allergies assessment [38]. Another study evaluated the ImmunoCAP and the EUROLINE system for the sensitization profiles towards egg white, yolk extract with the allergen components Gal d 1, 2, 3, 4. The authors determined that sIgE to Gal d 1 (single component) was highly specific in hen’s egg that affect corresponding allergic adults [39].

### 3.2. The Activation Test (BAT)

The BAT is a functional assay that measures the degree of degranulation of the basophils after stimulation with specific allergens, this being basophil reactivity (% CD63+ basophils) and basophil sensitivity (EC50), the main outcomes of the test [40]. Activation of basophils can be detected through upregulation of CD63 activation marker, then its expression is correlated with histamine released. EC50 is the concentration eliciting the half-maximal basophil activation and is a measure of basophil sensitivity [40]. The large number of articles published on BAT in the last year (2022) and the trend towards more regulation by the FDA, it is essential to understand methodological aspects also [41]. For example, passive sensitization experiments, selection of the optimal allergen concentration or the determination of the threshold. [42]. Considering these technical aspects, the BAT has limitations such as the presence of basophils that non-respond to the allergen. Peanut allergy studies (LEAP/LEAP-On studies) have reported that when BAT is performed for a single time, 14% of patients have unreactive basophils [43]. This non-responder status could be associated to transient changes in cell signaling proteins and can be reversed in different culturing conditions.

BAT in FA has already been studied extensively. In a peanut allergy study, the BAT for hazelnut, cashew nut, sesame, almond, peanut discriminated between allergic and non-allergic children, its sensitivity to peanut ranged between 96 and 100% [44]. A prospective study of patients from pediatric centers and universities, children between 0.5 and 17 years with confirmed allergy or sensitization to peanuts and/or tree nuts (almonds, cashews, hazelnuts, pistachios, walnuts) determined that BAT could predict allergic clinical status, and therefore may reduce the need for high-risk OFC in patients [45]. In addition, there was a study that also described these technique as a diagnostic tool in LTP allergy [46] showing that BAT could differentiate between LTP allergic patients and tolerant controls (Ara h 9), although neither the reactivity nor sensitivity could distinguish the severity of clinical symptoms. In a randomized controlled trial in children, it has been showed that BAT reduces the need for a food challenge test in children suspected of IgE-mediated cow’s milk allergy (CMA) [47]. The results showed that only a 37% reduction was achieved in in the requirement for food challenge. Similar results were obtained in a clinical trial for children with egg allergy, here BAT for egg allergy was considered a better diagnostic test than to double-blind placebo-controlled food challenge. The results showed a 41% reduction in the number of OFC was achieved [48].

### 3.3. Mast Cell Activation (MAT)

An alternative test to the BAT is the mast cell activation test (MAT). This consists of measuring the degranulation of mast cells, through levels of CD63, CD107a expression and the release of mediators (prostaglandin D2 and β-hexosaminidase) [49]. MAT is carried out in a similar way to BAT, although it is less sensitive [50]. In a study testing of peanut-sensitized patients (allergic patients) vs. non-peanut-sensitized patients (non-allergic patients), MAT provided conclusive results to aid in the diagnosis of allergic patients [51]. Related to peanut allergy, a study analyzed the activation of mast cells with the presence of Ara h 2 in a group of allergic children. MAT used for Ara h 2 strongly correlated with Arah2-sIgE levels, indicating specific mast cell response, and constituting an alternative diagnostic pathway [52]. Another study demonstrated for the first time the utility of MAT in the diagnosis of LTP allergy, with higher specificity compared to sIgE determination. The results from the study concluded that MAT can be used as complementary tool in the diagnosis of LTP allergy and just with BAT increased the sensitivity up to 95% [53].

### 3.4. T Cells Assay

The study of allergen-specific T cells is limited by the low frequencies of these cells in blood and the lack of methods able to characterize them. However, last year’s development of innovative techniques such as single-cell, genomic, epigenomic and immune repertoire sequencing [54] opened the door to progress the application of T cell assays as diagnostic tool for FA. CD8 T cells attenuate FA in some experimental models, while in humans, CD8 T cells have been shown to expand in response to wheat ingestion corresponding to celiac disease. Additionally, another study showed that CD8 T cells are activated by a peanut peptide in a dependent manner with peanut allergic individuals [55]. These CD8 T cells could express CCR4, suggesting that they were involved with a type 2 allergic immune response. A further study has identified a new Th2 effector, follicular subtypes with potential functional consequences in the pathogenesis and severity of allergic disease in patients with milk-triggered disease (EoE) [56]. A clinical trial identified characteristics of the peanut-specific CD4 T cell response in FA patients, correlated with high clinical sensitivity [57].

Therefore, theses in vitro diagnostic tools have been valuable to better understand the underlying mechanisms of allergic reactions to food and have helped to evaluate and develop treatments, which we will describe in the following sections.

**Table 1:** Diagnostic tests for FA including allergen-specific IgE, the basophil activation test, the mast cell activation test, and T cells assay.

Diagnostic Tools	FA	Advantages	Limitations
Specific IgE 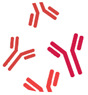	Sesame [32] Hazelnut [33] Peanut allergic children [35] Kiwi [36] LTP (Multiple Food) [38] Egg white and yolk [39]	Standardized High throughput Can be automatized	Poor in detection of sensitization from clinically reactive FA.
BAT 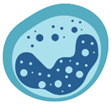	Peanut [44] LTP (Multiple Food) [45] Cow’s milk [47]	High sensitivity	Lack of standardization. Requires fresh blood. Its specificity is variable.
MAT 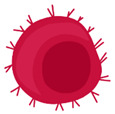	Peanut [51,52] LTP (Multiple Food) [53]	Requires plasma. Its specificity is stable.	Lack of standardization. Low sensitivity.
T cells 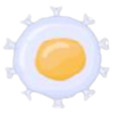	Peanut [55] Milk [56]	High throughput High sensitivity and specificity Concordant with clinical results	Need to improve the development of molecular techniques, and the characterization of allergens. Larger amounts of blood.

LTP: lipid transfers protein; FA: food allergy.

## 4. FA Immunotherapy Treatment

Immunomodulatory treatments in FA are therapeutic approaches that regulate the exaggerated immune response (Table 2). These treatments are in various stages of research and development, and some of these have been already used in clinical trials with promising results.

### 4.1. Allergen Immunotherapy

Allergen immunotherapy (AIT) has been shown to increase the reactivity threshold in most FA individuals [58]. Regarding this, different clinical trials have demonstrated that oral immunotherapy (OIT) for food allergens is safe and effective, improving the quality of life for peanut allergic patients. OIT for peanut has been approved by the FDA and EMA for its use in FA [9,59]. Peanut OIT is safe for children 1 to 3 years of age, from which 71% became desensitized, tolerating peanut protein [60]. Similar results were observed in patients (aged 7 to 55 years) treated with peanut OIT, highlighting that sustained unresponsiveness (SU) was only achievable in less than 35% of those who were successfully desensitized and the SU was maintained for a year [61]. It demonstrated that peanut OIT induces the blocking antibodies [62], low basophil activation and peanut-specific IgE [63].

Epicutaneous immunotherapy (EPIT) employs a non-invasive delivery system of the food allergen. Regarding EPIT, a clinical trial using peanut patches among peanut-allergic children, this assessed the efficacy and adverse events of EPIT. Its results provided a modest success, improving threshold sensitivity to one peanut (300 mg protein) for 35.3% after one year of therapy [64] and for patients after 130 weeks of desensitization using EPIT, this reached a threshold of 400 mg peanut protein [65].

Sublingual immunotherapy (SLIT) is another alternative to OIT and EPIT, which has proven to be safe and effective [66,67]. Peanut SLIT induced long-term desensitization in peanut allergic patients after 3 to 5 years of treatment. Recently, a study reported that during 16 weeks of SLIT with recombinant (r) Mal d 1, but not rBet v 1, significantly improved also for birch pollen-related apple allergy and showed that allergen-sIgE-blocking IgG antibodies were associated with clinical efficacy [68]. Although the SLIT treatment was conducted with only two patients, the results showed that SLIT was able to reduce the levels of antigen-sIgE in severe egg allergy also [69]. Furthermore, in a prospective study where patients diagnosed with LTP allergy and treated with Pru p 3 SLIT were included. One year after the start of Pru p 3 SLIT, the patients had negative OFC to peach and after 2 years of treatment, the OFC remained negative for walnuts and/or peanuts confirming the safety of the therapy [70].

Taking all these results into account and the importance of IgG antibodies in AIT, it has been proposed that IgG antibodies could have a modulatory role in FA. A study has described that FA is associated with increased levels of food-specific IgG and that they interfere with IgE interaction by regulating mast cell and basophil functions [71]. A study for children with suspected FA determined different patterns of sIgG in persistent (peanut) and transient (milk and egg) FA. The authors measured sIgG levels and sIgG isotypes regarding these foods, this showed that for peanut the sIgG, sIgG1, sIgG2, sIgG3 and sIgG4 were higher in peanut-allergic than in non-peanut-allergic patients. However, there is no difference in allergen-specific IgG isotypes when observed between allergic and non-allergic for milk or egg. With exception for milk-specific IgG4 that was higher in non-cows-milk-allergic than in cows-milk-allergic children [72]. They found no evidence that IgG was able to bind to receptors on the surface of mast cells or basophils or to suppress IgE-mediated activation of mast cells or basophils after allergen stimulation. sIgG4 is the most interesting of the IgG antibodies due to its inherent anti-inflammatory properties and its clinical relevance [73,74]. It has been reported that treatment with AIT in patients with peanut allergy, regulated the sIgG4 levels [75]

### 4.2. Nanoparticles: Platform AIT

Although the use of simple immunotherapies modify the immune response towards food tolerance, there are alternative strategies that promote desensitization such as the use of nanoparticles [11]. A large number of studies are investigating the safety and efficiency of the use of nanoparticle-encapsulated purified peanut extract in peanut allergic patients and animal models [76], masked administration of allergens (peanut) are encapsulated in poly(lactide-co-glycolide) (PLG). Nanoparticles attenuated the anaphylactic response in mouse models with peanut allergy, inducing a more tolerogenic phenotype and conferring protection from intragastric allergen challenge [77]. An innovative therapy has been developed based on the use of lipid nanoparticles (LNP) with encapsulated mRNA encoding peanut allergen epitopes. LNP demonstrated an increase in IL-10-producing Treg cells, suppression of Th2-mediated cytokine production, IgE synthesis and mast cell release in a peanut animal model [78,79].

Nanoparticles with T cells epitopes of arginine kinase with CpG attenuated shrimp allergen enhancing the FOXP3 expression and IL-10 production with a decrease in the Th2 differentiation [80]. Associated with CpG, another study showed that oral pre-treatment with β-lactoglobulin derived peptide and CpG co-encapsulated in PLG nanoparticles prior to sensitization, attenuates the development of CMA in mice [81,82].

New glycosystems functionalized with mannose or fucose and specific ligands combined with Pru p 3 peptides are focused on the modulation of the immune response via C-type lectin receptors (CLRs) [83,84] or Toll-Like Receptors (TLRs) [85]. Regarding this, glycosylated nanostructures combined with immunotherapy, induced long-lasting tolerance with specific transcriptional and methylations changes on DCs [86,87], Treg cells [88] in a peach allergy mouse model.

### 4.3. Hypoallergenic Proteins: Product AIT

The modification of allergens, through a physical or chemical alteration of their structures have been developed to improve the tolerance response, inducing Th1 and Treg responses. Within the hypoallergenic proteins, synthetic peptides and recombinant proteins have been designed as immunotherapy or vaccines to treat FA [11,89].

Allergy to cow’s milk requires the avoidance of cow’s milk proteins, currently the use of hypoallergenic milk protein formulation (CM-based hydrolysates or hydrolyzed rice) has been a strategy for the management of CMA [90,91,92]. The effects of these formulations have showed a tolerance response in human cells [93]. From the hydrolyzed formulas, the structural alteration of the Bos d 5 allergen (B5M) has been identified as a product for use in a hypoallergenic vaccine for CM allergy. B5M induced IgG antibodies and inhibited the degranulation of basophils induced by Bos d 5 [94].

Hypoallergenic derivatives of *Scylla-paramamosain* (mud crab), heat-stable tropomyosin (TM) and myosin light chain (MLC) have been preliminarily explored in vitro. A study recently showed that hypoallergenic derivatives of heat-stable allergens (mtTM, mtMLC) alleviated FA symptoms in a crab allergy model, inducing a significant IL-10 production which equilibrated Th1/Th2 cells [95]. Regarding shrimp allergy, TM has been modified by glycation (GTM) and combined with Al(OH)3 to form hypoallergenic complex and be used in AIT. This hypoallergenic complex has been able to induce desensitization in allergic reactions in shrimp allergic patients [96,97]. The glycation in the sesame proteins has been shown to reduce the allergenicity of sesame proteins effectively, identifying new hypoallergenic products to treat FA [98].

In addition, hypoallergenic wheat line (1BS-18H) lacking ω5-gliadin, induced oral tolerance to wheat gluten proteins in a wheat allergy rat model [99]. In this study, the results demonstrated that the early ingestion of 1BS-18H wheat before immunization induced oral tolerance to gluten and ω5-gliadin, a suppression of gluten-sIgE and IgG_1_ levels with an induction of Treg cells.

Although the recombinant Mad d 1 shows high allergenicity [100], recently a study showed that the recombinant Mal d 1 combined with immunotherapy blocked the IgE-mediated reactions also improving apple allergy [68].

### 4.4. Monoclonal Antibodies (Anti-IgE): Adjuvant AIT

Monoclonal antibodies (anti-IgE) have been considered as adjuvants in food AIT treatments [101] and as monotherapies [102]. These are based on the IgE neutralization. which reduces the sensitivity of the immune system to food allergens, reducing the activation of mast cells and basophils [103].

As monotherapy, an observational study has reported that patients with FA and severe asthma treated with omalizumab were able to increase the allergen threshold for milk, egg, wheat, hazelnut, also control of severe asthma. This resulted in an improvement in the quality of life [102]. An observational study which included children with severe CMA who did not respond successfully to OIT were treated with omalizumab. Interestingly, their CM threshold and IgG4 milk-specific protein levels were significantly increased [104]. Therefore, as monotherapy it can help patients to consume multiple foods and allow for increasing the dose of where its limited.

However, in recent years, several studies have revealed its role as adjuvant for AIT. A phase 2 randomized controlled multisite study using omalizumab combined with OIT decreased time to desensitization and simultaneously desensitizes multiple food allergens [105]. In addition, the combination of omalizumab with peanut OIT induced an increase in peanut intake, a reduction in sIgE, an increase in sIgG4 for peanut, Ara h 1 and Ara h 2 [106,107]. Another study similar determined that OIT with adjunctive anti-IgE can induce immunological changes decreasing type 2 immune response (IL-4 peanut-reactive CD4 T cells, downregulation of CD86 expression in antigen-presenting cell subsets and reduction in pro-inflammatory cytokines) [104]. Additionally, in a cohort of 181 patients, omalizumab dose-related efficacy in OIT was adjusted based on body weight, regardless of total IgE level [108]. Taken together, these results suggest that as an adjunct to OIT, omalizumab can facilitate rapid desensitization, regulate IgE and IgG4 and induce the change immunologically.

**Table 2:** Immunotherapeutic approaches for FA: advantages and limitation.

Immunotherapy	Treatment Type/FA	Advantages	Limitations
Allergen immunotherapy	OIT: Peanut [60,61,62,63] EPIT: Peanut [65] SLIT: Peanut, Apple, Egg and LTP (Multiple Food) [66,67,68,69,70]	-AIT is specific treatment that can address underlying cause of the allergic reaction. -Long-term effects, reducing the severity of allergic reactions. -ATI eliminates the need for other medications to control allergic reactions	-Lack of clinical studies to evaluate its safety and efficacy -Risks of suffering severe allergic reactions during treatment -Prolonged duration of treatment
Nanoparticles	PLG and LNP: Peanut [77,78,79] T cells epitopes + CpG: Shrimp and CM [80,81,82] Glycoparticles: Peach [86,87,88]	-Controlled delivery of allergens. -Improved absorption and bioavailability	-More studies are needed to evaluate their long-term safety and effectiveness -Technical complexity -Lack of regulation and approval for its implementation as therapies
Hypoallergenic proteins	Hydrolyzed protein formulation and B5M: CMA. [90,91,92,94] mtTM, mtMLC: Crab [95] GTM: Shrimp [96,97] 1BS-18H: Wheat [98,99] r Mal d 1: Apple [68]	-Reduced risk of allergic reactions.	-Potential nutrient deficiency -Cost and availability -Altered taste and texture
Monoclonal antibody (anti-IgE)	Monotherapy: Multiple Food and CM [102,104] Multiple therapy: Multiple Food and peanut [105,106,107]	-Reduced risk of allergic reactions (IgE inhibition). -Increasing food intake. -Improves the quality of life of patients.	-Cost -Side effects -Duration of treatment

AIT: Allergen immunotherapy; OIT: oral immunotherapy; EPIT: epicutaneous immunotherapy SLIT: sublingual Immunotherapy; PLG: poly(lactide-co-glycolide); LNP: lipid nanoparticle; CMA: cow´s milk allergy. r: recombinant.

## 5. New Therapeutic Approaches

Despite advances in specific treatments, they present certain limitations (Table 3); because of this, the interest in alternative medicine is increasing. Although there are not defined limits between these new natural approaches, we can classify them into three groups: probiotics, herbal medicine and dietary supplements.

### 5.1. Probiotics and Symbiotics

The microbiome has recently been described as a crucial interface between environmental factors and the development of FA, among other allergic diseases. This relationship has been exploited through the microbial immune modulatory effects of probiotics, prebiotics and symbiotics for the generation of food allergen tolerance, maintaining the Th1/Th2 cell balance, thus improving intestinal barrier function, controlling the intestinal microbiota and its metabolism [109].

The effect of lactic acid bacteria on food protein allergies in infants is studied most, showing a regulation of the intestinal flora of allergic infants, hydrolysis of allergens, inhibition of the inflammatory response, enhancement of the intestinal barrier and modulation of immune cell differentiation [110].

Several studies in FA animal models have demonstrated how some probiotics show the capacity to increase the ratio of effector Treg cells and enhance the secretion of regulatory cytokines (IL-10) such as *Clostridium butyricum* and *Lactobacillus gasseri* [111,112] including the probiotics from the *Bifidobacterium* species [113].

In recent study, the activation of the TLR4 pathway by *Bifidobacterium animalis* KV9 and *Lactobacillus vaginalis* FN3 also led to modulation of the Th1/Th2 balance, attenuating allergic responses in FA mice [114]. The study demonstrated that KV9 and FN3 possessed anti-allergic activities, they modulated the expression of IRF-1 and IRF-4.

Decreasing antigen-specific immunoglobulins has also been an important goal in probiotic therapies. In a murine model of CMA, 3 probiotics with anti-allergic properties have been identified, showing a reduction in the levels of IgE, IgG1, IgG2a, and β-lactoglobulin-specific mast cell protease [115]. In other ovalbumin (OVA)-induced FA animal models, the probiotic *Akkermansia muciniphila* BAA-835 attenuated the levels of IgE anti-OVA and eosinophils [116].

There are numerous interventional trials utilizing prebiotics, probiotics or symbiotics to investigate its effects on FA. In a randomized controlled trial based on the coadministration of probiotic (*Lactobacillus rhamnosus)* with peanut OIT, this reduced peanut-specific IgE levels and increased peanut-specific IgG4 [117]. In addition, the use of probiotic peanut OIT leads to substantial and continued improvement for quality of life 4 years after treatment [118,119]. This outcome has also been described with the combination of heat killed *Lactiplantibacillus plantarum* and OIT in CMA. In fact, this clinical trial showed improved tolerance to CM and an increase in the sIgG4 level with reduction in IL-5 and IL-9 [120].

A symbiotic-containing fructo-oligosaccharides and *Bifidobacterium breve* M-16V helped transform the gut microbial composition of non-IgE CM allergic infants to resemble that of healthy infants [121,122]. A pilot study with a symbiotic showed potential in the improvement of symptoms in infants with CMA; however, it lacked a proper a control group [123].

### 5.2. Herbal Medicine

In the past, herbal plants have been used for medical issues. Nowadays, many studies are being conducted to identify anti-allergic agents from these plants to treat IgE-mediated and non-IgE-mediated allergic reactions [124].

FA Herbal Formula 2 (FAHF-2), composed by 9 different herbs was the first plant origin-based drug approved by US FDA for FA treatment (2007). New techniques have been applied to improve some of its disadvantages. Two types of formulas have been purified, this being B-FAHF-2 (butanol purified FA herbal formula-2) and E-B-FAHF-2 (ethyl acetate and butanol purified FA herbal formula-2) [125]. Recently, in vivo experiments into murine models of peanut allergy showed that E-B-FHFA-2 and its active compound (berberine) protected the mice from anaphylaxis, decreased IgE levels and IgE plasma cells [126].

Apart from FAFH-2, in addition to its improved versions, many other plant origin compounds have been tested in vitro and in vivo for FA treatments. In this sense, N-nornuciferine and lirinidine alkaloids from lotus seed pods showed potent anti-food allergic activity in RBL-2H3 cells measured as β-hexosaminidase activity [127].

The oleuropein was evaluated for the prevention of OVA- induced FA. The results of their studies showed that sensitized mice treated with oleuropein had decreased levels of IgE, IgG and histamine. Moreover, oleuropein enhanced intestinal epithelium, altering mucosal mast cells and Treg cells [128]. Additionally, other compounds as the isoflavones isolated from kakkonto, a Japanese herbal medicine (Genistein and genistin) suppressed allergic symptoms in OVA-induced FA mice [129].

### 5.3. Dietary Supplements

Some components of food have been related to pro-inflammatory effects (fat, sugar, folate deficit) and others to anti-inflammatory effects (omega-3 PUFAs, vitamin D, polyphenols). Some studies have focused on diet supplementation as a treatment for FA disorders [130].

A study observed that ginger could upregulate the expression of the retinoic acid (RA) receptor in the gut, which suggested that immune responses mediated through RA regulation could contribute to the suppression of FA inflammation [131].

An OVA-sensitized mouse model supplemented with olive oil had a reduction in allergic symptoms, with a reduction in cytokines associated with Th2 cells and an increase in cytokines released by Treg cells [132]. The combined supplementation with arachidonic and docosahexaenoic acids during suckling period presented beneficial effects on OVA oral tolerance of the rat offspring, revealing that this combination decreased Th2 immune response due to an increase in Th1 cytokine levels [133]. A meta-analysis study concluded that omega-3 supplementation during pregnancy reduces the risk of FA in children. On the other hand, omega-3 supplementation during childhood did not show any beneficial effects [134].

**Table 3:** Therapeutic approaches and their achievements.

Therapeutic Approaches	Model/FA	Achievements
Probiotics and symbiotics	*Clostridium butyricum, Lactobacillus gasseri* and Bifidobacterium species [111,112,113] and *Lactobacillus vaginalis* [114]: FA animal model. *Lactobacillus paracasei* L9: CM [135] *Leuconostoc citreum*: FA animal model [116] *Akkermansia muciniphila*: FA animal model [136]. *Lactobacillus rhamnosus:* Penaut [117] Synbiotic-containing fructooligosaccharides and *Bifidobacterium breve* M-16V:CM [121,122].	-Increase the ratio of effector Treg cells and enhance the secretion of regulatory cytokines. -Modulation of Th1/Th2 balance and attenuation of allergic reaction. -IgE reduction -Transformation of gut microbial to improve the healthy infants.
Herbal medicine	E-B-FAHF-2 and B-FAHF-2: Peanut [125,126]. Berberine: peanut and cholera toxine animal model [126]. Oleuropein: FA animal model [128].	-Reduction in anaphylaxis symptoms. -Reduction in histamine and IgE plasma levels. -Reduction in B cells in spleen and modification of gut microbiota
Dietary supplements	Ginger: CACO2 cells [131]. Olive oil: FA animal model [132]. Arachidonic and docosahexaenoic acids (PUFAs): mother during suckling period levels [133]. Omega-3 supplementation: mothers during pregnancy [134].	-Suppression of FA inflammation. -Reduction in allergic symptoms. -Th2 cells reduction and increase in Treg. -Increase in oral tolerance in children. -Increase in Th1 cytokines levels. -Reduction in the risk of FA in children.

FA: food allergy; CMA: cow´s milk allergy.

## 6. Conclusions

FAs are complex and mediated by a variety of immunological mechanisms. The IgE-mediated response is the most common, but cellular mechanisms and other types of immunological reactions can also be involved. Understanding these mechanisms is essential for the proper diagnosis and treatment of FA. In this sense, it is essential to note that diagnostic tools continue to develop constantly, since they have certain limitations, such as needing to be agreed upon or the low sensitivity. Nonetheless, theses in vitro diagnostic tools have helped to evaluate and develop new treatments for FA.

Regarding immunomodulatory approaches as treatment for FA, many of them are still in the research and development stages. However, all of them are focused on reducing the allergic response by inducing a tolerance response. Despite this, they still present certain weaknesses that give rise to new therapeutic approaches. More studies are still needed to investigate the role of IgG in FA.

Overall, new therapeutic approaches for the prevention and treatment of FAs have shown clinical potential; however, the results of these trials remain controversial. With contradiction, these approaches face several limitations including the heterogeneity in dosages/administration in trials and the mechanism of probiotics or herbal medicine for FA remains unknown. Further studies are required to discern the actual benefits of these new therapies in FA.

## Data Availability

Not applicable.

## Abbreviations

ATI	Allergen immunotherapy
BAT	Basophil activation test
CMPA	Cow milk protein
EGID	Eosinophilic gastrointestinal disorders
EoE	Eosinophilic esophagitis
EMA	European Medicines Agency
FA	Food allergy
FPIES	Food protein-induced enterocolitis syndrome
FPIAP	Food protein-induced allergic proctocolitis
FPE	Food protein-induced allergic enteropathy
FDA	Food and Drug Administration
GORD	Gastro-esophageal reflux disorder
MAT	Mast cell activation test
NSAIDs	Nonsteroidal anti-inflammatory drugs
OFC	Oral food challenge
OIT	Oral immunotherapy
SPT	Skin prick test
SMPA	Soja’s milk protein allergy
sIgE	Specific immunoglobulin E
TSLP	Thymic stromal lymphopoietin
